# A Novel Ivermectin-Derived Compound D4 and Its Antimicrobial/Biofilm Properties against MRSA

**DOI:** 10.3390/antibiotics10020208

**Published:** 2021-02-20

**Authors:** Xinyi Tan, Haoji Xie, Bin Zhang, Jiale Zhou, Zhende Dou, Xiao Wang, Ning Wang

**Affiliations:** 1 Immunology Innovation Team, School of medicine, Ningbo University, Ningbo 315211, Zhejiang, China; 186001806@nbu.edu.cn (X.T.); 186001827@nbu.edu.cn (H.X.); 186001867@nbu.edu.cn (J.Z.); 2 Li Dak Sum Yip Yio Chin Kenneth Li Marine Biopharmaceutical Research Center, College of Food and Pharmaceutical Sciences, Ningbo University, Ningbo 315800, Zhejiang, China; zhangbin1@nbu.edu.cn (B.Z.); 1711091025@nbu.edu.cn (Z.D.); 3 Institute of Drug Discovery Technology, Ningbo University, Ningbo 315211, Zhejiang, China

**Keywords:** ivermectin derivative, MRSA, antibacterial, antibiofilm, mechanisms

## Abstract

Methicillin-resistant *Staphylococcus aureus* (MRSA) and its biofilms infection is still a serious threat to global health. It is urgent to develop efficient drugs by repositioning or designing drugs to solve this problem. In this study, the antibacterial/biofilm activity and mechanisms of ivermectin (D) and its 4″-position amino substitution derivative (D4) against MRSA were investigated. The minimum inhibitory concentration (MIC) of D was 20 μg/mL, which is four times higher than D4 (MIC = 5 μg/mL). The mechanism research demonstrated that D4 was more potent than D at destroying bacterial cell wall, permeating cell membrane (6.25–36.0% vs 1.92–6.04%) and binding to MRSA genomic DNA. Moreover, after incubation with 10–40 μg/mL D4 for 24 h, the percentages of biofilm decreased by 21.2–92.9%, which was more effective than D (no significant change at 40 μg/mL). The antibiofilm effect is achieved by regulating the expression of related genes (*RSH*, *relQ*, *rsbU*, *sigB*, *spA*, and *icaD*). Additionally, though the higher hemolysis makes D4 a safety risk for intravenous injection, other administration options could be considered as well. Therefore, all the results have indicated that D4 may be a potential candidate compound for the treatment of MRSA and its biofilm infections.

## 1. Introduction

Methicillin-resistant *Staphylococcus aureus* (MRSA) infection is still a serious threat to global health [1]. The diseases caused by MRSA, such as bacteremia, endocarditis and sepsis, are associated with poorer clinical outcomes [2]. Meanwhile, the formation of biofilms also increases its resistance to antimicrobial drugs and host defenses by forming communities and lowering metabolism [3]. *S. aureus* biofilms could attach to indwelling medical devices, including implanted artificial heart valves, catheters and joint prosthetics, then leading to fatal infectious disease [4]. All these infections are characteristically chronic and frequently occur in hospitals [5]. Vancomycin was the first-line agent for management of hospitalized patients with MRSA infections [6]. However, the adverse effects [7] and slowly growing prevalence of vancomycin-resistant *S. aureus* (VRSA) are becoming two worrying features for its future use [8]. Thus, it is urgent to develop new drugs to solve this problem.

Gradually, drug repositioning receives widespread attention. Existing drugs are expected to be applied to new therapeutic areas [9]. Although ivermectin (D) has been extensively used as an antiparasitic drug, it has a proved antimicrobial activity against *S. aureus* in vitro [10]. Moreover, due to the well-known pharmacology and toxicology of ivermectin [10], as well as its certain quorum-sensing and biofilm inhibitory activity [11,12], it has the potential to be a new drug. However, on the one hand, its antibacterial mechanism has never been reported. On the other hand, even its maximum plasma concentration cannot reach its antibacterial concentration. A single oral dose between 100 and 200 μg/kg of ivermectin is recommended for treating various types of parasitic diseases. When people follow a single oral dose of 150 μg/kg, the maximum plasma concentration is 52.0 ng/mL [13], which is far less than the minimum inhibitory concentrations (MIC) of ivermectin against MRSA. The value of its MIC is 12.5 μg/mL [10]. This may be the reason why ivermectin is not used as an antimicrobial drug to cure diseases caused by MRSA.

Therefore, we try to change its chemical structure and physicochemical properties to improve its antibacterial activity. Since ivermectin is a macrocyclic lactone and most antibacterial macrocyclic lactones contain basic amines [14], amino-containing drugs generally have good water solubility and are easy to generate salt under physiological conditions, which is beneficial to improve the absorption, distribution, excretion and metabolism of drugs. Previous research has demonstrated that the introduction of an amino group into the carbon chains of carbohydrate could significantly improve its bioactivity [15,16,17]. Additionally, 4″-position is easier and more suitable for introducing amino substituent than other sites [14]. Hence, in our study, after the substitution of 4″-position hydroxyl group by amino, a novel ivermectin-derived compound was designed. Then the antimicrobial/biofilm activities and antibacterial mechanisms of D and D4 against MRSA were investigated.

## 2. Materials and Methods

### 2.1. Bacterial Strains and Cell Lines

D (Ivermectin, Purity ≥98%) was purchased from Shanghai Macklin Biochemical Co., Ltd. (Shanghai, China). Methicillin-resistant *S. aureus* (MRSA) ATCC 43300 were purchased from the American Type Culture Collection (ATCC) (VA, USA). The RAW 264.7 cells were donated by Dr. Guo Hua (Ningbo University, Ningbo, China).

### 2.2. The Preparation of 4-amino-4-deoxyivermectin B1 (D4)

**Figure 1:**
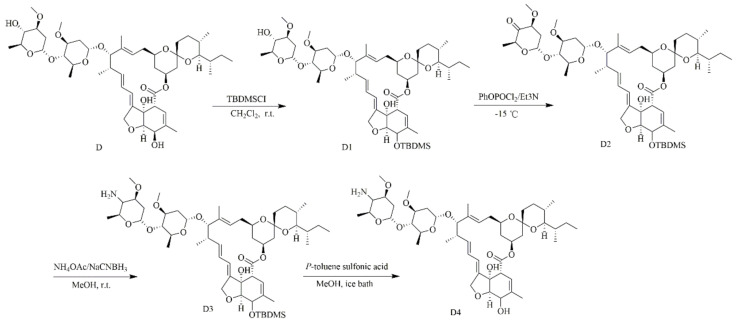
The four-step synthetic route of D4.

D4 was prepared via a four-step synthetic method, as reported in the literature [18]. Instead of using avermectin B1 as the starting material in the literature, this study replaced it with D. The synthesis route (Figure 1) was briefly described as follows: First, the 5-hydroxyl group of D was selectively protected with tertbutyldimethylsilyl chloride (TBDMSCl) to obtain the intermediate 5-O-TBDMS-ivermectin B1 (D1), followed by the oxidation of 4″-hydroxyl of D1 under the PhOPOCl_2_/Et_3_N system in dry dimethyl sulfoxide to give 4″-oxo-5-O-TBDMS-ivermectin B1 (D2). Next, 4″-NH_2_-5-O-TBDMS-ivermectin B1 (D3) was obtained by reductive amination with NH_4_OAC/NaBH_3_CN. Finally, a tertbutyldimethylsilyl protecting group of D3 was removed by *p*-toluene sulfonic acid to obtain the target compound D4. The structure of D4 was established via ^1^H NMR, ^13^C NMR and high-resolution mass spectral data.

### 2.3. Antimicrobial Activity

#### 2.3.1. Minimum Inhibitory Concentration (MIC)

The MIC values of D and D4 against MRSA ATCC 43300 were determined by the microtiter broth dilution method. The bacteria were grown to mid-logarithmic phase and 1 × 10^5^ CFU/mL cells were diluted into the Mueller Hinton (MH) broth. Two-fold serial dilutions of D and D4 were prepared (40 to 0.078 μg/mL). A total of 2 μL D, D4 and dimethyl sulfoxide (DMSO) (as a solvent control) and 98 μL bacterial suspensions were added into 96-well plates. Then the plate was incubated for 16–24 h at 37 ℃. Vancomycin was tested as control. All assays were performed in triplicate. The MICs were determined by examining visible bacterial growth with naked eyes [19].

#### 2.3.2. Time-Kill Curves 

To determine the effects of D and D4 on growth curves, mid-log phase MRSA ATCC 43300 was diluted to 1 × 10^5^ CFU/mL with fresh medium. Then bacteria solution (5 mL) and different concentrations of D and D4 (1×, 2×, 4×MIC) were added to a 50 mL shaking flask. DMSO and vancomycin at 2×MIC were used as negative and positive control. Subsequently, the mixtures were cultured at 37 °C and 250 rpm. A total of 150 μL samples were taken from each flask at 0, 0.5, 1, 2, 4, 6, 8, 10, 12 and 24 h. The number of bacteria was measured by plate colony count. All tests were run in triplicate [20]. 

### 2.4. Hemolysis and Cytotoxicity

#### 2.4.1. Hemolysis

To evaluate hemolytic activity of D and D4, the hemoglobin released from healthy mouse red blood cells was determined after treatment with these two compounds. Blood cells were washed and collected by centrifugation at 1500 rpm for 5 min. An equal volume of red blood suspensions (8%, *v/v*) and different concentrations (0.3125–160 μg/mL) of compounds were mixed. DMSO and 0.1% Triton X-100 served as negative and positive controls. Subsequently, the mixtures were incubated at 37 °C for 1 h and centrifuged at 1500 rpm for 5 min. Finally, absorbance of supernatants was measured at 540 nm. Hemolysis (%) = [(OD540 nm of the treated sample-OD540 nm of the negative control) / (OD540 nm of positive control-OD540 nm of negative control)] ×100 %. Three replicates were performed for each condition [21]. 

#### 2.4.2. Cytotoxicity

Cell Counting Kit-8 (CCK-8) assay was performed to determine the effect of D and D4 on the viability of murine RAW264.7 macrophage cells. RAW264.7 cells (2.5 × 10^4^ cells/well) were added into 96-well plates and incubated at 37 °C in a humidified 5% CO_2_ environment for 24 h. Then various concentrations (0.15–20 μg/mL) of D and D4 were mixed with cells. DMSO was used as a control. Finally, 10 µL WST-8 solutions were added to each well. After incubating for 4 hours at 37 °C, the absorbance of each sample was measured by a microplate reader at 460 nm. The following formula was used to calculate cell viability: Cell viability (%) = OD 460 nm of treated sample/OD 460 nm of control × 100 [22].

### 2.5. Effects of D and D4 on Cell Wall and Membrane

#### 2.5.1. Scanning/Transmission Electron (SEM/TEM) Microscope Observations

MRSA ATCC 43300 (1 × 10^8^ CFU/mL) in mid-log phase were treated with 4×MIC D and D4 at 37 °C for 2 h. Then 2.5% glutaraldehyde was used to fix bacteria at 4 °C for 12 h. For SEM observation, the bacteria were dehydrated with 20%, 50%, 70%, 85%, 95% and 100% ethanol solutions and dried at room temperature overnight. Gold-palladium was sputtered on samples. The images were captured by S4800 SEM. For TEM observation, 1% OsO4 was used to post-fix the bacteria, 50%, 70%, 85%, 95% and 100% acetone were used to dehydrate the samples. Then, they were immersed in epoxy resin and embedded in capsules containing embedding medium, polymerized at 45 °C for 3 h and at 65 °C for 24 h, respectively. Ultramicrotome was used to acquire thin sections, followed by staining with 1% uranyl acetate. Images were visualized by a Hitachi H-7650 TEM [20].

#### 2.5.2. Membrane Permeabilization Analysis 

To investigate the bacterial cell membrane permeabilization activity of D and D4, the propidium iodide (PI) uptake assay was carried out. Mid-log phase MRSA ATCC 43300 (1 × 10^8^ CFU/mL) were incubated with 1×MIC, 2×MIC and 4×MIC D and D4 solutions for 5, 30 and 120 min at 37 °C, respectively. The bacteria without treatment were used as negative controls. After washing twice with PBS, all samples were incubated with 50 μg/mL PI for 15 min. Finally, the fluorescence was analyzed by FACS Calibur Flow Cytometer (BD, USA) [21].

### 2.6. Effects of D and D4 on Bacterial Genomic DNA

#### 2.6.1. Gel Retardation Assay

The interaction of compounds and MRSA genomic DNA was examined by gel migration assay. Bacterial genome extraction kit was used for obtaining genomic DNA. A series of two-fold dilution (12.5 to 400 μg/mL) compounds and DNA were mixed and incubated at room temperature for 10 min. The migration of genomic DNA was analyzed by electrophoresis on a 0.8% agarose gel.

#### 2.6.2. Circular Dichroism (CD) Spectroscopy

To further investigate the secondary structure changes of MRSA ATCC 43300 genomic DNA, CD spectra were measured after treatment with D and D4. Genomic DNA (150 μg/mL) and compounds (200 μg/mL) were mixed and incubated for 10 min at room temperature with a DMSO-treated sample as a negative control. Then a 1.0-mm path length cuvette was used to load the samples. Finally, the spectra (230–320 nm) were recorded at 25 °C with a 10 nm/min scanning speed by J-1700 CD spectrometer [23]. 

### 2.7. Ability of D and D4 against MRSA Biofilms

#### 2.7.1. Effects on Biofilm Formation 

Mid-logarithmic phase MRSA ATCC 43300 (1 × 10^8^ CFU/mL) was grown in 96-well plates with tryptic soy broth (TSB) medium at 37 °C for 24 h in the presence (1.25–40 μg/mL) or absence of D and D4. Fresh TSB medium was used as a negative control. After removing planktonic bacteria, the biofilms were stained with 0.1% crystal violet for 30 min. Then the samples were rinsed with PBS twice, the dye binding to biofilm was resolubilized in 95% ethanol. A microplate reader was used to measure the absorbance at 570 nm [24,25].

#### 2.7.2. Biofilms Observed by SEM

To further explore the inhibition ability of D and D4 to the formation of MRSA ATCC 43300 biofilms. A concentration of 1 × 10^8^ CFU/mL mid-log phase bacteria was seeded into 24-well plates with a silicon slice in each well. Then D (80 μg/mL) and D4 (20 μg/mL) were added and the mixture was incubated for 24 h. After washing with PBS to remove the planktonic bacteria, the biofilm could be observed by SEM after immobilization, dehydration, drying and coating [20].

#### 2.7.3. Effects on Transcription of Biofilm Formation-Related Genes

Biofilm formation related genes *RSH*, *relP*, *relQ*, *rsbU*, *sigB*, *spA*, *AgrA* and *icaD* were chosen in our study with *16s rRNA* as a housekeeping one. Primer sequences were listed in Table 1. MRSA ATCC 43300 was incubated with 4×MIC D and D4 for 2 h. Then the bacteria were washed with PBS, total RNA was isolated and cDNA was obtained after removing genomic DNA. A real-time reverse transcription-polymerase chain reaction (qRT-PCR) was carried out at last. The relative expression ratios were calculated as the following formula: n-fold transcription = 2^−△△Ct^, △△Ct = △Ct (drug-treated)/△Ct (untreated), in which △Ct represents the difference between the cycle threshold (Ct) of the gene studied and the Ct of housekeeping 16s rRNA gene (internal control). Student’s *t* test was used for analyzing the results [26].

### 2.8. Statistical Analysis

All data were analyzed by GraphPad Prism 6 and presented as mean ± SD (standard error of the mean). One-way ANOVA or student’s *t* test were used for comparisons among multiple groups. A *p*-value of <0.05 was considered statistically significant.

## 3. Results

### 3.1. The Characterization of 4-amino-4-deoxyivermectin B1 (**D4**)

Four-step total yield: 6.6%; white solid; mp:157–160 °C; ^1^H NMR (600 MHz, Chloroform-*D*): δ 5.86 (d, *J* = 10.4 Hz, 2H, 1H, H9), 5.77–5.68 (m, 2H, H10, H11), 5.44–5.31 (m, 3H, H3, H1″, H19), 5.01–4.96 (m, 1H, H15), 4.78–4.75 (m, 1H, H1′), 4.71–4.63 (m, 2H, H8a), 4.29 (d, *J* = 4.7 Hz, 1H, H5), 4.02–3.92 (m, 3H, 7-OH, H6, H13), 3.82 (dd, *J* = 9.5, 6.2 Hz, 1H, H5′), 3.63 (ddd, *J* = 49.5, 4.8, 2.7 Hz, 3H, H17, H5″), 3.40 (d, *J* = 32.4 Hz, 6H, H3′, H25, H3″, 3′-OCH_3_), 3.30–3.19 (m, 3H, 3″-OCH_3_), 3.03 (dd, *J* = 3.5, 1.6 Hz, 1H, H4′), 2.51 (m, 1H, H12), 2.50–2.23 (m, 5H, H16, H24, H2′), 2.01 (m, 1H, H20), 1.83 (s, 3H, 4-Me), 1.77 (d, *J* = 8.8 Hz, 1H, H18b), 1.66–1.37 (m, 10H, H20, H26, H27, H2″, H22, H23), 1.50 (s, 3H, 14-Me), 1.27–1.11 (m, 9H, 6′-Me, 6″-Me, 12-Me), 0.94–0.79(m, 10H, 27-Me, 24-Me, 26-Me, H18a); ^13^C NMR (151 MHz, DMSO-*d*_6_): δ 170.6, 162.8, 141.2, 136.0, 135.4, 134.8, 125.0, 119.7, 118.3, 118.0, 97.5, 96.9, 94.4, 81.2, 81.0, 79.5, 78.7, 77.5, 75.4, 68.4, 67.8, 66.9, 66.7, 66.7, 66.5, 56.5, 55.7, 45.5, 41.3, 38.5, 35.9, 35.0, 34.8, 34.7, 34.0, 33.5, 30.7, 27.7, 26.6, 19.9, 19.4, 18.5, 18.1, 17.7, 17.2, 14.5, 12.2, 12.0; HRMS: *m/z* calcd for C_48_H_76_NO_13_ [M+H]^+^: 874.5311, found: 874.5313.

### 3.2. Antimicrobial Activity

#### 3.2.1. MIC Determination

As shown in Table 2, MIC of D and D4 against MRSA ATCC 43300 was 20 and 5 μg/mL. D4 displayed more potent antibacterial activity against test bacteria compared to parental compound D, while the antimicrobial activity of them was still less than that of vancomycin (MIC = 1 μg/mL). 

#### 3.2.2. Time-Killing Curves 

**Figure 2:**
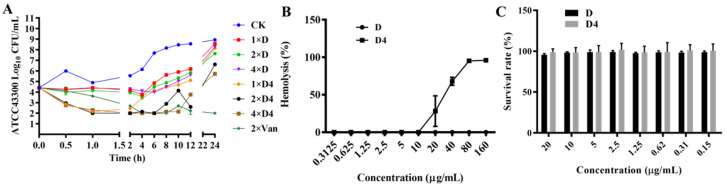
The extracellular killing curves, hemolysis and cytotoxicity of D and D4. (**A**) Bactericidal kinetics assay of D, D4 and vancomycin against MRSA ATCC 43300. (**B**) The hemolysis of D and D4 against the red blood cells of mice. (**C**) The cytotoxicities of D and D4 against the RAW 264.7 cells.

Time-killing curves of D and D4 were showed in Figure 2A. Bacteria amount of MRSA ATCC 43300 was significantly decreased within 0.5 h after treatment with 1×, 2×, and 4×MIC of D4, which indicated that the antimicrobial efficiency of D4 was superior to D and vancomycin. At the concentration of 1×, 2×, and 4×MIC, D could only inhibit bacterial growth for 4 h. The antibacterial activity of 2× and 4×MIC D4 could last for 6–10 h, which is longer than D. When compared to the parental compound, we found that D4 not only had faster bactericidal activity in the early stage but also had a longer lasting effect.

### 3.3. Hemolysis and Cytotoxicity

#### 3.3.1. Hemolysis

The hemolysis of D and D4 to erythrocytes was determined. As shown in Figure 2B, hemolytic activities of D4 to murine erythrocytes at the concentrations of 20, 40, 80 and 160 μg/mL were 28.159%, 67.953%, 95.140% and 95.955%, respectively. However, the hemolysis of D was 0% at these concentrations, which was obviously lower than D4. These results indicated that the modification of D in our study enhanced the activity and hemolysis at the same time. However, D4 does not impair the integrity of red blood cells at effective concentrations.

#### 3.3.2. Cytotoxicity

A CCK-8 assay was used to evaluate the cytotoxicity of D and D4 against murine RAW264.7 macrophage cells. The results elucidated that cell survival rate when incubated with D and D4 at the concentration of 20 μg/mL was 95.506% and 98.916% (Figure 2C), respectively. These data illustrated that these two compounds have a very low cytotoxicity activity against the test cells.

### 3.4. Effects of D and D4 on Cell Wall and Membrane

#### 3.4.1. Scanning/Transmission Electron (SEM/TEM) Microscope Observations

**Figure 3:**
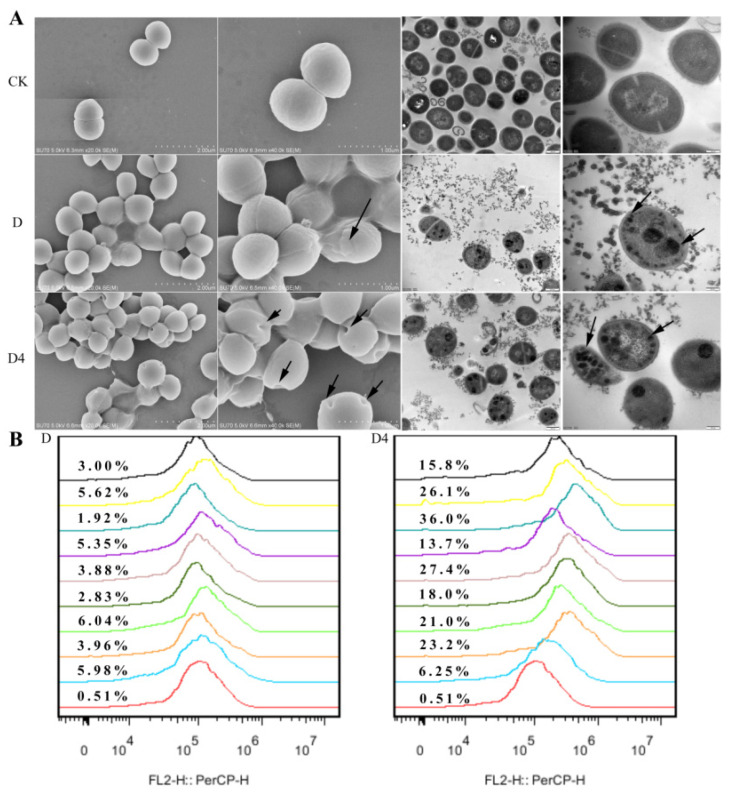
Effects of D and D4 on cell wall and membrane. (**A**) Scanning electron microscopy and transmission electron microscopy analysis of MRSA ATCC 43300 cells treated with D and D4. (**B**) Flow cytometric analysis of PI-staining in MRSA ATCC 43300 cells treated with 1×, 2× or 4×MIC D and D4 for 5, 30 or 120 min, respectively. Red line: no compound, negative control; Blue line: treatment with 1×MIC compounds for 5 min; Orange line: treatment with 2×MIC compounds for 5 min; Green line: treatment with 4×MIC compounds for 5 min. Bottle-green line: treatment with 1×MIC compounds for 30 min; Brown line: treatment with 2×MIC compounds for 30 min; Purple line: treatment with 4×MIC compounds for 30 min. Blue-green line: treatment with 1×MIC compounds for 120 min; Yellow line: treatment with 2×MIC compounds for 120 min; Black line: treatment with 4×MIC compounds for 120 min.

SEM was used to directly observe the change in morphology, integrity and cellular structure of MRSA ATCC 43300. After incubated with 4×MIC for 2 h, about 20% shrunken and bubbling bulges bacteria cells were observed in the D-treated group. In the group treated by D4, holes and disruptions were found on nearly 80% of the bacterial surface, which was more serious than the D group. Normal intact cell morphology was observed in the untreated control group (Figure 3A). Additionally, the internal ultrastructure image of MRSA ATCC 43300 was captured by TEM. After exposure to D, trachychromatic speckled aggregates appeared in the bacteria. Except for this phenomenon, severe damage to bacterial cell walls, cell membranes and cytoplasm were observed in the D4-treated group too. Deformed cell morphology, cellular contents leakage and ghost bacteria displayed in the image, which indicated a better performance of D4. 

#### 3.4.2. Membrane Permeabilization Analysis 

Nucleic acid fluorescent dye PI was used to evaluate the effects of D and D4 on the bacterial cell wall and membrane. PI can penetrate the damaged bacterial cell membranes and then the density of the bacteria could be determined by flow cytometry. As shown in Figure 3B, the fluorescence rate of the untreated group was 0.51%, which indicated that the bacterial cell membrane was intact before treatment. After incubation with 1×, 2× and 4×MIC of D and D4 for 5 min, 30 min and 120 min, the percentages of PI-permeable bacteria were 1.92–6.04% and 6.25–36.0%, respectively. The results illustrated that the novel compound D4 had a stronger penetrating ability than D.

### 3.5. Effects of D and D4 on Bacterial Genomic DNA 

#### 3.5.1. Gel Retardation Assay

**Figure 4:**
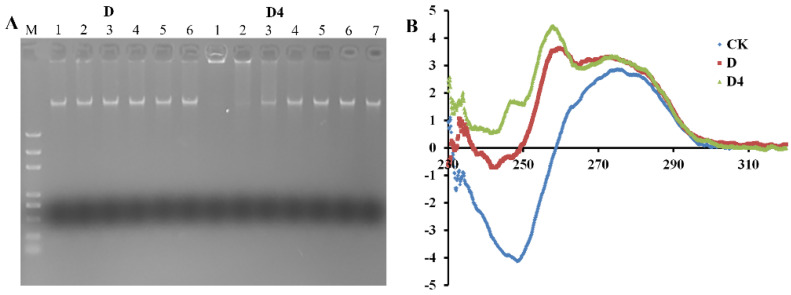
Interaction of D and D4 with MRSA ATCC 43300 bacterial genomic DNA. (**A**) Interaction of D and D4 with bacterial genomic DNA by a gel migration assay. M: DNA marker; 1–6: The concentration of compounds was 400, 200, 100, 50, 25 and 12.5 μg/mL, respectively; 7: Control. (**B**) CD spectra of genomic DNA from MRSA ATCC 43300 in the presence of D and D4. The concentration of compound and DNA were 200 and 150 μg/mL, respectively.

DNA-binding properties of D and D4 were evaluated by a genomic DNA gel retardation assay. D could not inhibit the migration of MRSA ATCC 43300 genomic DNA at the test concentrations. However, the movement of DNA could be restrained by D4 when the concentration was up to 100 μg/mL (Figure 4A). This result indicated that D4 could bind to bacterial genomic DNA, and the binding efficiency was higher than that of the parental compound.

#### 3.5.2. CD Spectroscopy

The binding affinity of D and D4 with MRSA ATCC 43300 genomic DNA were further detected by CD spectrometer. DNA morphology change was supervised after incubation with the compounds. Normal MRSA ATCC 43300 genomic DNA showed a negative and positive peak at 280 and 250 nm in the CD spectrum (Figure 4B). After treatment with D and D4, the negative peak decreased sharply (D), even disappeared (D4). Another positive peak was found in the image, which indicated the changes in DNA helical structure. The destructive potential of D4 against DNA conformation was higher than D, which was consistent with the gel retardation assay.

### 3.6. Ability of D and D4 against MRSA Biofilms

#### 3.6.1. Inhibition of Biofilm Formation

**Figure 5:**
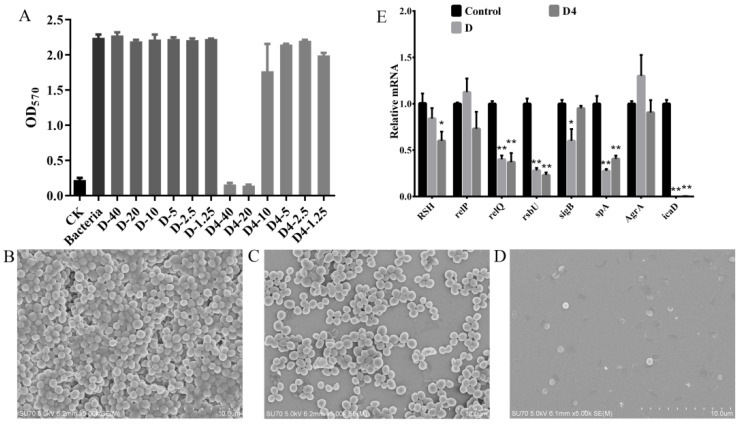
The abilities of D and D4 against MRSA biofilms. (**A**) Inhibition effect of D and D4 on biofilm formation. (**B**–**D**) Observation of MRSA ATCC 43300 biofilms by SEM; (**B**) Untreated biofilm’ (**C**) D-treated biofilm, (**D**) D4-treated biofilm. (**E**) Relative gene expressions of biofilms. MRSA ATCC 43300 cells were incubated with 4×MIC compounds or no compound for 2 h. The transcriptional levels of biofilm formation related genes were detected by qRT-PCR. All assays were performed in triplicate. The analyses were measured by one-way ANOVA, with Duncan’s multiple comparisons test. A probability value of <0.05 was considered significant. (*) Indicates the significance between control and each of treatment groups. * *p* < 0.05; ** *p* < 0. 01. The results are given as the mean ± SD (*n* = 3).

A crystal violet staining assay was used to evaluate the MRSA ATCC 43300 biofilm formation inhibition ability of D and D4. The results were shown in Figure 5A, D4 inhibited biofilm formation in a concentration-dependent manner. The percentages of biofilm decreased by 92.9%, 93.6% and 21.2% when the bacteria were treated by 40, 20 and 10 μg/mL of D4 for 24 h. However, D had no effect on biofilm formation at the highest test concentration (40 μg/mL), indicating that the inhibition ability of D4 against MRSA ATCC 43300 biofilm formation was remarkably higher than that of D.

#### 3.6.2. Inhibition of Biofilms Observed by SEM

To further confirm the inhibition ability of D and D4 against biofilm formation, the 64 μg/mL compound-treated bacteria were observed by SEM. As shown in Figure 5B–D, the untreated group MRSA ATCC 43300 formed a thick biofilm on the surface of silicon slice. D4 treatment completely inhibited the biofilm formation with only several damaged bacterial cells retained on the slice. However, D had a lesser inhibition ability to the bacteria attachment. Though the biofilm was thinner than the control group, almost all the bacteria still presented normal morphologies. All these results indicated that the anti-biofilm activity of D4 is remarkably superior to D.

#### 3.6.3. Effects of D and D4 on the Transcription of Biofilm Formation Related Genes

After treatment with 4×MIC D and D4 for 2 h, the MRSA ATCC 43300 mRNA transcription levels of biofilm formation related genes were determined. The results indicated that the transcription of *relQ*, *rsbU*, *spA* and *icaD* genes were only 0.37–0.40, 0.23–0.28, 0.27–0.405 and 0.0004–0.00155 fold to the control level, which were significantly decreased by the incubation with D and D4. The transcription of *sigB* genes in the D-treated group were 0.60 time downregulated, while the transcription of the *RSH* gene (0.60 fold to the control group) was only significantly inhibited by D4 (Figure 5E). These observations suggested that D and D4 could inhibit the formation of biofilms by regulating the transcription of related genes in MRSA ATCC 43300.

## 4. Discussion

Although antibiotics are the main drugs to fight against pathogenic bacteria, and some new drugs are also being developed to target resistant bacteria, the drugs available to kill resistance bacteria are still limited. *S. aureus* resistance to methicillin is now widely described, and thus the development of new drugs is urgently necessary [27]. *S. aureus* are also known to form biofilms, the multi-layered community of bacteria that is difficult to eradicate, leading to treatment failure and recurrent episodes of infections, such as catheter-associated infections, wound infections and UTIs [28,29,30,31]. As an FDA-approved anti-parasitic previously, D warrants further investigation for possible benefits in humans. In this study, we designed a novel compound based on D by amino substitution at 4″-position (Figure 1) to improve its antibacterial/biofilm activity, then the mechanism was also explored.

Previous studies have confirmed that D has an anti-bacterial effect against certain *S. aureus* isolates. In our study, the conclusion was further proved, though MIC was about 2-fold higher, which may be due to the difference in *S. aureus* strain [10]. However, the time-kill kinetics data indicated that the effect of D was probably bacteriostatic rather than bactericidal, which was consistent with the research of Ashraf et al [10]. As anticipated, after a 4″-position modification, the novel compound D4 has a 4-fold higher antimicrobial activity than D (Table 2). The possible explanation for this is that the amino group could provide a positive charge, which has the ability to promote electrostatic attraction with the negatively charged bacterial cell membrane. Moreover, the amino group could also be used as a hydrogen bond donor, which is more conducive to the combination of drug molecules with the amino acid residues of target protein, thus enhancing the antimicrobial activity of the compound [32]. Further work will be necessary to confirm these explanations.

However, hemolysis of D4 was also enhanced at the same time with the improvement of antimicrobial efficiency, though the hemolytic concentration was 4-fold higher than MIC (Figure 2B). This result suggests that intravenous is not the appropriate choice for administration of D4, while oral or topical administration can be tried for this novel compound because of its low cytotoxicity at the highest test concentration (20 μg/mL) (Figure 2C). In our study, the maximum investigation dose of D and D4 was limited by the concentration of the solution and the cytotoxicity of DMSO. Considering the changes of biological properties, the activity mechanism of D4 needs to be further explored, thus paving the way for designing more efficacious analogues. 

To our knowledge, this study is a maiden attempt, reporting the antimicrobial mechanism of D and its derivative D4 against MRSA. Firstly, the wrinkled bacteria in D group and the holes on the surface of D4-treated bacteria indicated that these two compounds could destroy the cell wall to varying degrees, which conquered the bacterial first line of defense. Subsequently, entered PI indicated that D4 can kill bacteria by interacting with and permeabilizing bacterial cytoplasmic membranes, thus leading to leakage of contents and the aggregation of an intracellular matrix (Figure 3A). With the increase of treatment time and compound concentration, the general trend of fluorescence rate increased first and then declined (Figure 3B), which is consistent with a previous report [23]. This phenomenon suggested that the pores were formed on the cell wall and membrane of bacteria after treatment with a low dose of D4 for a short time. While the bacteria could be lysed by incubation with a longer time and a higher concentration of D4, which was confirmed by the TEM images. Subsequently, the fragments are not able to be detected with flow cytometry. Compared to D4, D is less effective in penetrating bacterial cell membranes. Thus, we deduced that the increased activity of D4 against MRSA ATCC 43300 was associated with the electrostatic interaction of the amino group with the bacteria cell wall and membrane, which is consistent with the view of a previous report which demonstrated that positive charge was extremely important for the activity of antibacterial compounds due to their ability to promote electrostatic attraction with the negatively charged membrane of microorganisms [32]. 

Subsequently, according to the aggregation of the intracellular matrix, the DNA binding affinity of D and D4 was further investigated. D4 appears to have a higher blocking ability to MRSA ATCC 43300 genomic DNA than D, which may also benefit from the positively charged amino group to interact electrostatically with negatively charged DNA. This DNA-binding property is similar to some reported antibacterial compounds which could insert DNA base pairs and change its conformation [21,23]. However, it cannot explain the aggregation phenomenon of D-treated bacteria. Therefore, the target intracellular molecules and other mechanisms need to be further explored.

Additionally, planktonic MRSA ATCC 43300 could attach to niche surfaces and embed in extracellular substances, and then form biofilms. Most bacteria, including *S. aureus*, exist as biofilms rather than planktonic cells during infections [33]. Biofilms are usually more resistant to antimicrobials and often cause refractory diseases [34]. Previous reports have demonstrated that D exert weak and limited effects on *Acinetobacter baumannii* biofilms [33]. Therefore, we tested the anti-biofilm effects and mechanisms of D and D4. D4 was found to be more potent than D at inhibiting the formation of MRSA ATCC 43300 biofilms. One of the possible explanations for this is that D4 had an enhanced ability to kill the bacteria, and hence, fewer amounts of bacteria gathered to form biofilms. On the other hand, to explore the deep mechanism underlying bactericidal phenomena, the transcription of biofilm formation-related genes was evaluated.

According to the speculation of Yamabe et al., macrolides (including D) can be remote signals in bacterial quorum sensing systems, which is a main affect factor of biofilm formation [33]. The quorum-sensing system-related genes were detected. Firstly, our observation emphasized that the signaling molecule of the quorum sensing system (p)ppGpp metabolism-related genes *RSH*, and *RelQ* were significantly decreased, especially in the D4 group. This suggested that D4 could inhibit the synthesis of (p)ppGpp and then weaken the adaptation ability of bacteria to stressful environments [35]. Additionally, the agr quorum-sensing system is associated with bacterial adhesion. Hence, the related genes (*rsbU*, *AgrA* and *SigB*) were also detected in this study. Significantly reduced transcription of the *rsbU* gene was observed compared to the control group (Figure 5). The *rsbU* gene encodes a positive regulator of the alternative sigma factor B (SigB), which has a negative effect on the *AgrA* gene [36]. However, we noted that the expression of the *SigB* and *AgrA* gene was only reduced and enhanced in the D-treated group, while the change in the *AgrA* gene was not significant. Therefore, we deduce that D4 may not exert an antibiofilm activity by elevating extracellular protease and murein hydrolase levels, which are controlled by *AgrA* gene [36]. Moreover, staphylococcal protein A (SPA) and polysaccharide intercellular adhesion (PIA) encoded by the *spa* gene and ica operon, is considered one of the vital proteins involved in the attachment and biofilm formation of *S. aureus*. *icaD* is the most prevalent biofilm-forming gene among ica locus (icaADBC operon), which plays important role in exopolysaccharides synthesis [37,38]. *spA* and *icaD* were significantly downregulated by incubation with D and D4 (Figure 5), which indicated that the inhibition of biofilm forming is achieved by a decreased production of PIA and SPA. Subsequently, the levels of metabolites, such as protein or polysaccharide, coded by these genes need to be measured in further research.

## 5. Conclusions

In conclusion, the antimicrobial activity of D against *S. aureus* was further confirmed in this study. A novel compound D4 was designed based on D through 4″-position amino substitution. D4 was found to be more potent than D at destructing the bacterial cell wall, permeating cell membrane and binding to DNA. Therefore, D4 exhibited higher antimicrobial activity against MRSA. Additionally, D4 could also inhibit the biofilm formation of MRSA by regulating the expression-related genes. This study paves a way for drug repositioning and novel compound designing against bacterial and its biofilm-related infections. Moreover, the in-depth antibacterial mechanism and the in vivo effects need to be further studied.

## Figures and Tables

**Table 1:** Design of biofilm formation related genes primer.

Gene	Sequence (5′ to 3′)
*RSH-F*	TACATCGCACTGATTGCCCA
*RSH-R*	TTAAATTGCCGGCTGTCGAG
*relP-F*	TTGCCGGAATTCGCGTAGTA
*relP-R*	CGCGTTCTGCTAAAAAGACTGG
*relQ-F*	AGAAAGTGGTTACCGCTCGT
*relQ-R*	TCATCCGGATAAGCACCATCA
*rsbU-F*	CGCGTGAAGATGTGTTCAAGAC
*rsbU-R*	CTATCTCTTTATCGTGAACTTGAAG
*sigB-F*	GGTGCCATAAATAGATTCGATATGTCCTT
*sigB-R*	CTTTTGATTTCACCGATTACAGTAGGTACT
*spA-F*	GCGCAACACGATGAAGCTCAACAA
*spA-R*	ACGTTAGCACTTTGGCTTGGATCA
*AgrA-F*	AAGCATGACCCAGTTGGTAACA
*AgrA-R*	ATCCATCGCTGCAACTTTGTAGA
*icaD-F*	ATGGTCAAGCCCAGACAGAG
*icaD-R*	AGTATTTTCAATGTTTAAAGCAA
*16s rRNA-F*	GCTGCCCTTTGTATTGTC
*16s rRNA-R*	AGATGTTGGGTTAAGTCCC

**Table 2:** The MICs of D, D4 and vancomycin against MRSA ATCC 43300.

Drugs	MIC (μg/mL)
	ATCC 43300
D	20
D4	5
vancomycin	1

## Data Availability

All data generated or analyzed during this study are included in this published article and its supplementary information files.
